# Chemokine CXCL12 activates dual CXCR4 and CXCR7-mediated signaling pathways in pancreatic cancer cells

**DOI:** 10.1186/1479-5876-10-68

**Published:** 2012-04-02

**Authors:** Eileen L Heinrich, Wendy Lee, Jianming Lu, Andrew M Lowy, Joseph Kim

**Affiliations:** 1 Department of Surgery, City of Hope Comprehensive Cancer Center, 1500 East Duarte Road, Duarte, CA 91010, USA; 2 Department of Surgery, Moores Cancer Center, University of California San Diego, La Jolla, CA 92093, USA

**Keywords:** CXCR4, CXCR7, CXCL12, MAPK, pancreatic cancer

## Abstract

**Background:**

Previously assumed to be a select ligand for chemokine receptor CXCR4, chemokine CXCL12 is now known to activate both CXCR4 and CXCR7. However, very little is known about the co-expression of these receptors in cancer cells.

**Methods:**

We used immunohistochemistry to determine the extent of co-expression in pancreatic cancer tissue samples and immunoblotting to verify expression in pancreatic cancer cell lines. In cell culture studies, siRNA was used to knock down expression of CXCR4, CXCR7, K-Ras and β-arrestin -2 prior to stimulating the cells with CXCL12. Activation of the mitogen-activated protein kinase pathway (MAPK) was assessed using both a Raf-pull down assay and western blotting. The involvement of the receptors in CXCL12-mediated increases in cell proliferation was examined via an ATP-based proliferation assay.

**Results:**

First, we discovered frequent CXCR4/CXCR7 co-expression in human pancreatic cancer tissues and cell lines. Next, we observed consistent increases in ERK1/2 phosphorylation after exposure to CXCL12 or CXCL11, a CXCR7 agonist, in pancreatic cancer cell lines co-expressing CXCR4/CXCR7. To better characterize the receptor-mediated pathway(s), we knocked down CXCR4 or CXCR7, exposed the cells to CXCL12 and examined subsequent effects on ERK1/2. We observed that CXCR7 mediates the CXCL12-driven increase in ERK1/2 phosphorylation. Knockdown of CXCR4 expression however, decreased levels of K-Ras activity. Conversely, KRAS knockdown greatly reduced CXCL12-mediated increases in ERK1/2 phosphorylation. We then evaluated the role of β-arrestin-2, a protein directly recruited by chemokine receptors. We observed that β-arrestin-2 knockdown also inhibited increases in ERK1/2 phosphorylation mediated by both CXCR4 and CXCR7. Finally, we investigated the mechanism for CXCL12-enhanced cell proliferation and found that either receptor can modulate cell proliferation.

**Conclusions:**

In summary, our data demonstrate that CXCR4 and CXCR7 are frequently co-expressed in human pancreatic cancer tissues and cell lines. We show that β-arrestin-2 and K-Ras dependent pathways coordinate the transduction of CXCL12 signals. Our results suggest that the development of therapies based on inhibiting CXCL12 signaling to halt the growth of pancreatic cancer should be focused at the ligand level in order to account for the contributions of both receptors to this signaling pathway.

## Background

It is well-established that chemokines interact with G protein-coupled receptors (GPCRs) to activate downstream signaling pathways that enhance cancer cell growth, migratory behavior, and cell survival [1,2]. Previous studies have characterized the effects of chemokine CXCL12 in many cancers [3-5] including its role in promoting local invasion and distant metastasis of pancreatic cancer [4,6-8]. Its corresponding receptor CXCR4 has been widely investigated initially because reports showed it is a co-receptor for T-tropic HIV-1 and HIV-2 entry into CD4^+ ^cells [9,10]. Since then, CXCL12 was found to be the specific ligand for CXCR4 [11,12]. As such, the CXCL12-CXCR4 axis has been the focus of research into therapeutic strategies for pancreatic and other cancers [7,13-15]. Recent data, however, shows that CXCL12 also binds to and activates chemokine receptor CXCR7 [16-19]. Therefore, downstream cell functions, which have been previously attributed to CXCR4, may also result from CXCR7-mediated signaling.

CXCR7 is expressed in many different tissues, including neurons, immune cells, and endothelial cells; receptor-mediated signaling can occur by binding one of its two known ligands, CXCL11 or CXCL12 [17,18,20,21]. It has a dedicated role in fetal cardiac development and B-cell localization as elucidated in CXCR7-deficient mice [17,18,20,21]. As with many other chemokine receptors, CXCR7 is known to induce oncogenic phenotypes apart from its innate role in organogenesis and immunity [17,18,20,21]. Similar to what is known about CXCR4, recent reports have indicated that CXCR7 promotes cancer cell survival through anti-apoptotic mechanisms [17,22]. However, in contrast to the downstream effects of CXCR4, chemotaxis has not been reported to be induced by CXCR7-mediated signaling [17]. Although these data may suggest divergent functions for CXCR4 and CXCR7 in cancer cells, little is known regarding the frequency of co-expression and therein the mechanism for propagation of CXCL12 signals.

We previously investigated CXCL12 signaling in pancreatic cancer cells and observed enhanced cell proliferation mediated by the MAPK pathway [23,24]. Here, our objective was to investigate the roles of CXCR4 and CXCR7 in CXCL12-driven activation of the MAPK pathway in human pancreatic cancer cells. We examined β-arrestins which are recruited by GPCRs [25], as well as K-Ras, which is known to regulate the MAPK pathway [26]. Our results demonstrate that CXCR4 and CXCR7 are co-expressed with high frequency in human pancreatic cancers and that either receptor can regulate the MAPK pathway. Our results suggest that both CXCR4 and CXCR7 are potential targets in the development of effective therapies to halt the growth of pancreatic cancer.

## Materials and methods

### Cell culture and reagents

Human pancreatic cancer cell lines AsPC-1, MiaPaCa-2, PANC-1, SU.86.86, HS-766 T and BxPC-3,were obtained from American Type Culture Collection (ATCC; Manassas, VA) within the past 5 years. FG cells, which are a metastatic derivative of the pancreatic adenocarcinoma cells COLO-357, were provided by Dr. A. Lowy. All cells used for the experiments presented in this study were immediately cryopreserved in liquid nitrogen after they were obtained. All cell lines were assessed by DNA extraction, polymerase chain reaction (PCR) amplification, and sequencing for KRAS and TP53 gene mutations to verify the genotype of cells (data not shown). Cells were maintained in ATCC-recommended media at 37°C and 5% CO_2_. Serum-starvation lasted for 12-24 h unless otherwise noted.

CXCL12 and epidermal growth factor (EGF) were purchased from Peprotech (Rocky Hill, NJ); CXCL11and CXCL10 from R&D Systems (Minneapolis, MN). The following antibodies were used: rabbit polyclonal antibodies against phospho-ERK1/2 and total ERK1/2 (Cell Signaling; Beverly, MA), rabbit polyclonal antibodies against CXCR4 (Abcam, Cambridge, MA) and CXCR7 (Abcam and R&D Systems), a goat polyclonal antibody against β-arrestin-2 (Abcam), and a mouse monoclonal antibody against K-Ras (Calbiochem; San Diego, CA).

### Immunoblotting

Cell lysates were collected as previously described [27]. Twenty micrograms of protein were separated on 12% SDS-polyacrylamide gels and transfered onto PVDF membranes (Millipore; Bedford, MA). The membranes were blocked for 1 h and probed overnight with primary antibodies. After washing, membranes were labeled with horseradish peroxidase (HRP)-conjugated secondary antibodies (BioRad; Hercules, CA). Blots were developed with a chemiluminescence substrate (Amersham Pharmacia; Piscataway, NJ) and imaged.

### Tissue staining

We assessed CXCR4 and CXCR7 expression in formalin-fixed paraffin embedded (FFPE) specimens as previously described [27]. Pancreatic cancers were obtained from patients who had undergone resection for pancreatic adenocarcinoma with Institutional Review Board approval. Tissue blocks were sectioned (5 μm) and deparaffinized with xylene. After antigen retrieval was performed, a section was incubated with the anti-CXCR4 antibody and the next consecutive section from the tissue block was incubated with the anti-CXCR7 antibody. Then, they were labeled with secondary antibody (EnVision Plus; Dako, Carpinteria, CA), developed, and examined under microscopy at 200× magnification.

### Short interfering RNA (SiRNA)

Pancreatic cancer cells were transfected with siRNA (100 nM) using RNAiMAX (Invitrogen; Carlsbad, CA) according to the manufacturer's instructions and incubated for 48 h prior to application of treatments. siRNAs used were control, CXCR4, CXCR7, β-arrestin-2 and KRAS (Dharmacon; Lafayette, CO).

### K-ras activity

K-Ras activity was measured by a Raf pull-down assay (Millipore). In this enzyme-linked immunosorbent assay, cells maintained in serum-free media, were exposed to CXCL12 (100 ng/ml) or CXCL11 (200 ng/ml) for 15 min and then lysed. The cell lysate (100 μg) was incubated with Raf-1 Ras Binding Domain (RBD)-agarose. K-Ras proteins captured by Raf-1-RBD were detected and measured by the addition of an anti-K-Ras antibody (Millipore). An HRP-conjugated secondary antibody was then added. After adding a chemiluminescent substrate, signals were measured by a luminometer (Perkin-Elmer; Shelton, CT). Baseline K-Ras activity prior to stimulation with CXCL12 and CXCL11 was placed at zero; the data presented represents the relative increases in K-Ras activity due to stimulation. At least three independent assays were performed for each cell line. The mean absorbance ± one SD was plotted for each treatment group.

### Cell proliferation

Cell proliferation was assessed using a proliferation assay (CellTiter-Glo, Promega; Madison, WI) based on the quantification of ATP as previously described [27]. Cells were plated in 96-well plates at a density of 5 × 10^3 ^cells per well and exposed to CXCL12 in serum-free media for 72 h. Plates were incubated with CellTiter-Glo reagent and luminescence was measured. At least three independent cell proliferation assays were performed. Baseline proliferation prior to stimulation with CXCL12 and CXCL11 was placed at zero and the results show the relative increases due to stimulation. The mean absorbance ± one SD was plotted for each treatment group.

#### Statistics

Statistical analysis of the data was performed using unpaired Student's *t*-test. *P *values were two-sided and values of < 0.05 were considered statistically significant.

## Results

### CXCR4 and CXCR7 are co-expressed in human pancreatic cancers

**Figure 1:**
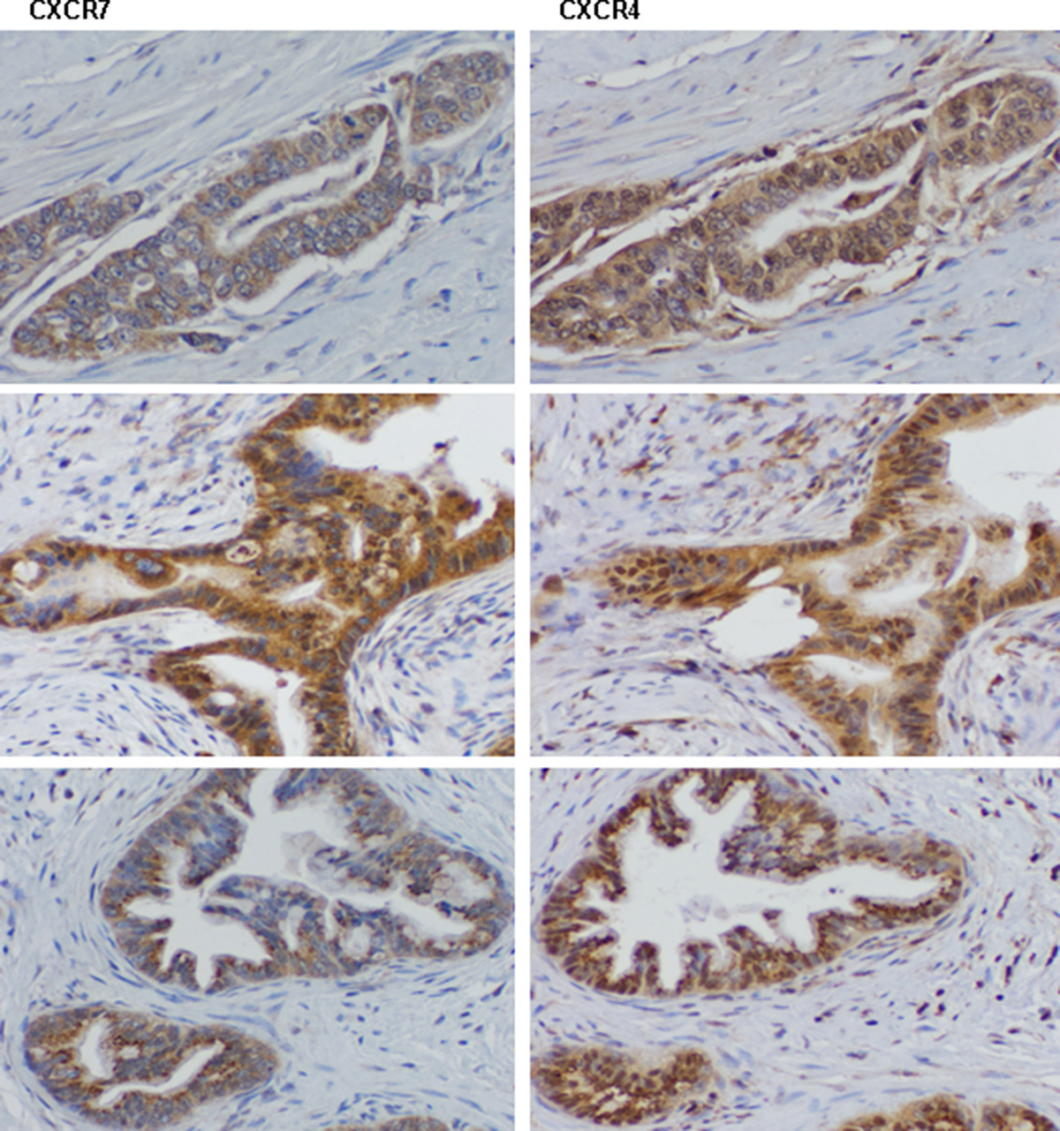
**CXCR4 and CXCR7 expression in human formalin-fixed paraffin-embedded pancreatic cancer specimens**. Pancreatic cancer tissue was obtained from patients who had undergone resection for pancreatic adenocarcinoma with Institutional Review Board approval. Tissue blocks were sectioned (5 μm) and deparaffinized with xylene. Antigen retrieval was performed and one section was incubated with the anti-CXCR4 antibody and the next consecutive section from the same tissue block was incubated with the anti-CXCR7 antibody.

To assess the clinical frequency of CXCR4 and CXCR7 co-expression, we performed immunohistochemical (IHC) staining in 51 FFPE human pancreatic cancer specimens. IHC demonstrated high frequency of CXCR4 and CXCR7 co-expression in these samples: 37 showed double staining, 5 showed single staining, while 9 had no staining. Representative IHC of CXCR4 and CXCR7 expression for three different patient samples are presented in Figure 1. For each patient sample, the section stained with the CXCR4 antibody and the corresponding CXCR7 section are immediately adjacent slices of the FFPE tissue. We have previously shown the absence of receptor staining in normal human pancreatic tissue samples with an increase in staining intensity over tumor stage [24].

**Figure 2:**
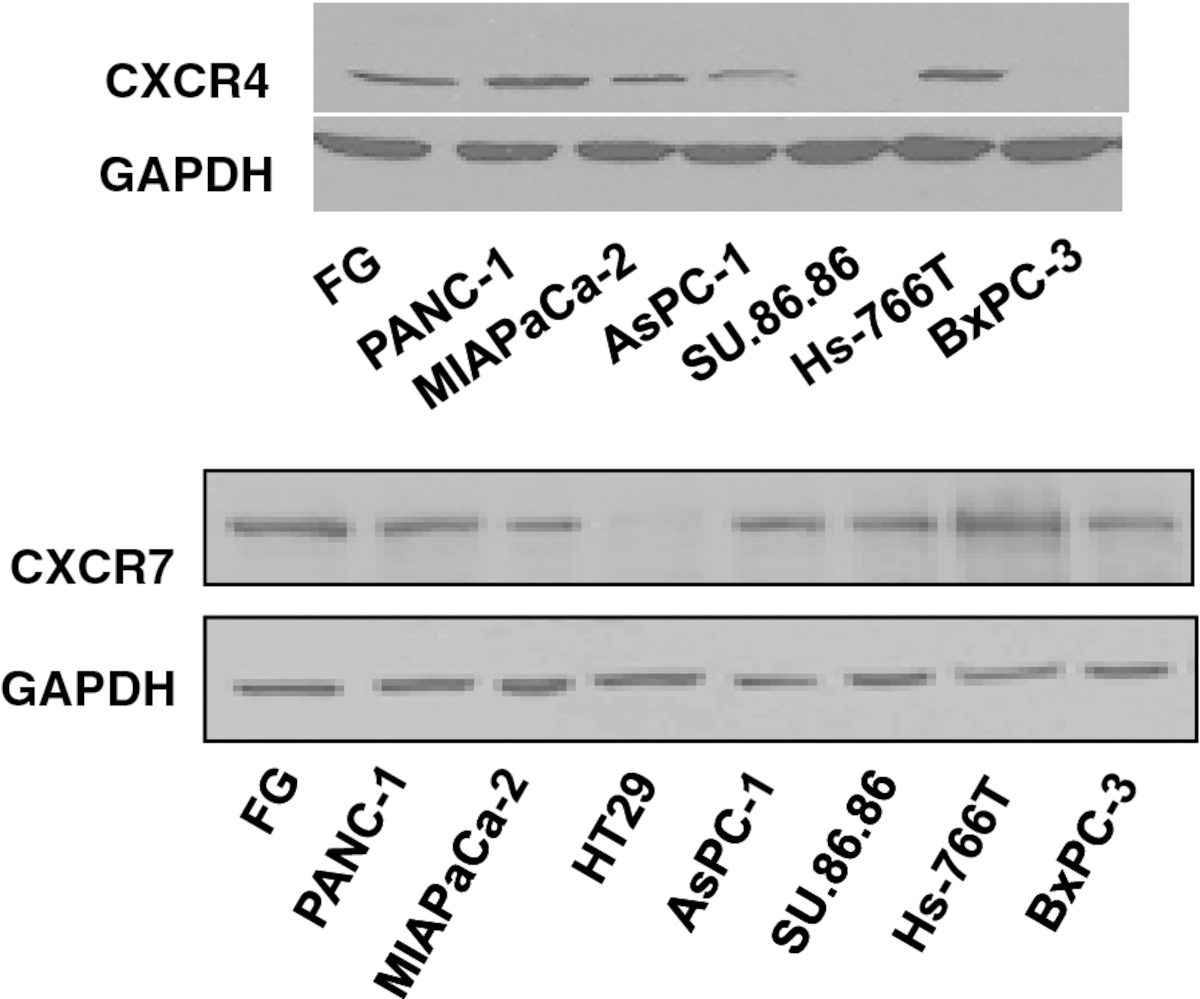
**CXCR4 and CXCR7 expression in pancreatic cancer cell lines**. Immunoblotting was performed for CXCR4 and CXCR7 protein expression in established human pancreatic cancer cell lines. HT29 colon cancer cells were used as a negative control for CXCR7 expression. GAPDH was used as a loading control.

CXCR4 and CXCR7 expression was assessed in 7 pancreatic cancer cell lines by immunoblotting. All cell lines expressed both CXCR4 and CXCR7, except for BxPC and SU.86.86 which lack CXCR4 (Figure 2). Our results showing no CXCR7 expression in HT29 colon cancer cells, which were utilized as a negative control, are consistent with published reports [22,28]. We selected PANC-1, MiaPaCa2, and FG cells for further investigation.

### CXCR4 and CXCR7 mediate the activation of the MAPK pathway

**Figure 3:**
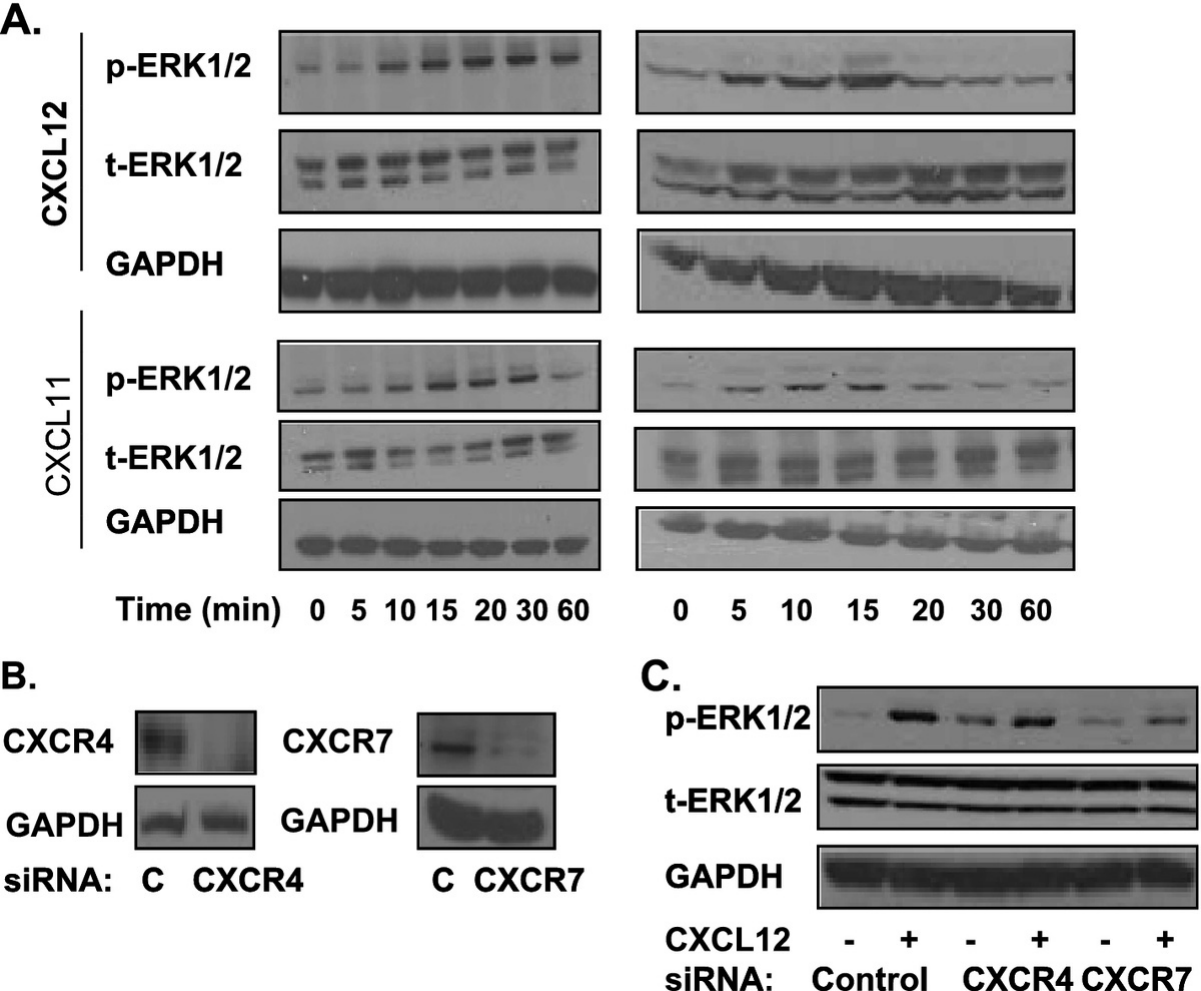
**The effect of CXCL12 exposure on MAPK signaling**. a) Pancreatic cancer cells PANC-1 and MiaPaCa2 were serum-starved and then exposed to CXCL12 (100 ng/ml) and CXCL11 (200 ng/ml) over a range of time periods (5 min to 1 h). Immunoblotting was performed for phosphorylated ERK1/2. Total ERK1/2 and GAPDH were used as loading controls. b) PANC-1 cells were transfected with control, CXCR4, or CXCR7 siRNA (100 nM) using RNAiMAX (10 μl). The immunoblots verify down-regulation of CXCR4 and CXCR7 expression. c) Following transfection, the cells were serum-starved and then exposed to CXCL12 (100 ng/ml). Immunoblotting was performed for phospho-ERK1/2. Total ERK1/2 and GAPDH were used as loading controls.

We exposed PANC-1 and MiaPaCa2 cells to CXCL12, which induced an increase in ERK phosphorylation in agreement with our previous results [26]. Then, through the use of CXCL11, we demonstrated that CXCR7-mediated signaling alone can also increase ERK phosphorylation (Figure 3a). By using CXCL12 and CXCL11, we show that both CXCR4 and CXCR7 can mediate ERK phosphorylation in cells co-expressing these receptors.

To further elucidate which receptor(s) mediate CXCL12-driven ERK phosphorylation, we performed CXCR4 or CXCR7 knockdown in PANC-1 cells (Figure 3b). Following knockdown, cells were exposed to CXCL12 (Figure 3c). CXCR7 knockdown was required to attenuate CXCL12-driven ERK phosphorylation. Our results suggest that CXCR7-mediated signaling can regulate CXCL12-driven ERK phosphorylation in cells co-expressing CXCR4/CXCR7.

### CXCR4 signaling is required for the CXCL12-induced increase in K-ras activity

**Figure 4:**
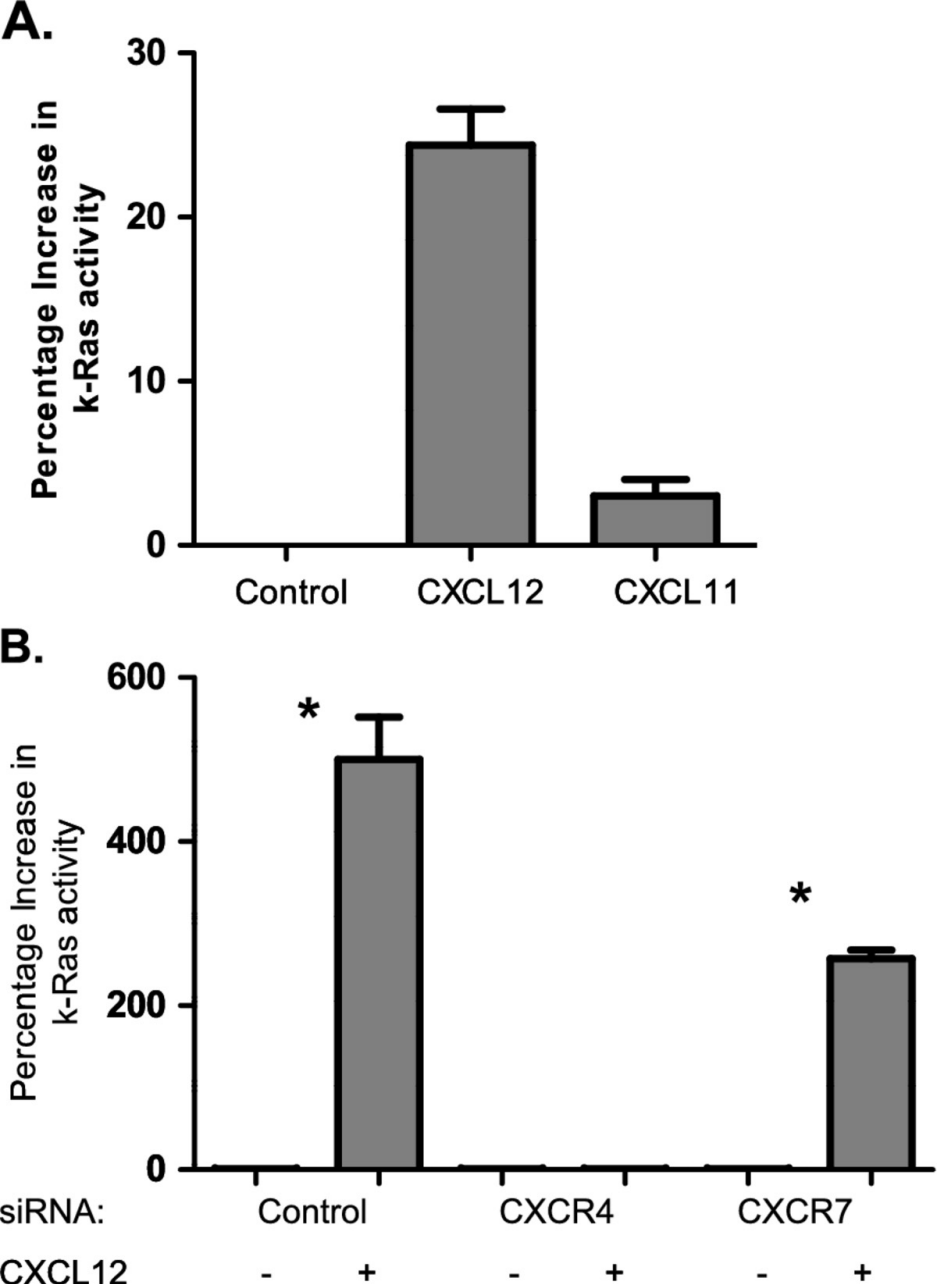
**K-Ras activity in response to CXCL12 exposure**. a) Pancreatic cancer cells were serum-starved and then exposed to CXCL12 (100 ng/ml) and CXCL11 (200 ng/ml) for 15 min. Whole cell lysates were assessed for K-Ras activity using a Raf pull-down assay. b) Cells were transfected with control, CXCR4, or CXCR7 siRNA (100 nM). Following serum-starvation cells were treated with CXCL12 (100 ng/ml) for 15 min. K-Ras activity was normalized to each untreated baseline level; and relative increases are depicted. The mean absorbance ± one SD was plotted for each treatment group. * designates *p *< 0.05.

We next sought to determine whether CXCR4 or CXCR7 signaling targeted K-Ras, a known regulator of the MAPK pathway. Utilizing a Raf pull-down assay, we observed increased K-Ras activity in PANC-1 cells following exposure to CXCL12 but not to the CXCR7 ligand CXCL11 (Figure 4a). To further validate CXCR4 as the receptor involved in the CXCL12-driven change in K-Ras activity, we again knocked down CXCR4 and CXCR7 expression in the cells prior to CXCL12 stimulation. We observed that CXCR4 knockdown, but not CXCR7, blocked CXCL12-induced changes in K-Ras activity (Figrue 4b). This supports our finding in Figure 4a that stimulating CXCR7 alone does not activate K-ras activity. We concluded that CXCL12-driven increases in K-Ras activity are mediated by CXCR4.

**Figure 5:**
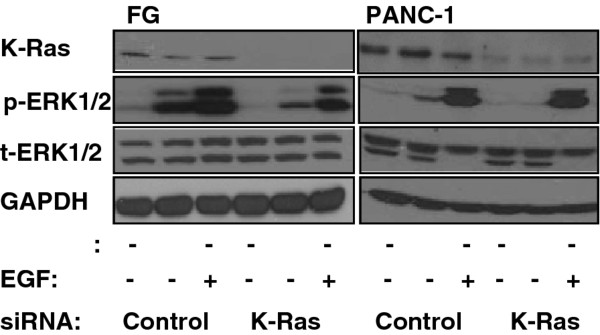
**The effects of KRAS knockdown on ERK phosphorylation following CXCL12 treatment**. Pancreatic cancer cells, FG and PANC-1 (left and right immunoblots respectively) were transfected with control or KRAS siRNA (100 nM). Cells were serum-starved and then exposed to CXCL12 (100 ng/ml) or EGF (100 ng/ml) for 15 min. Immunoblotting was performed for K-Ras and phospho-ERK1/2. Total ERK1/2 and GAPDH were used as loading controls.

To directly assess the role of K-Ras in CXCL12 signaling, we knocked down KRAS using siRNA and then exposed pancreatic cancer cell lines to CXCL12. We observed that KRAS knockdown greatly reduced CXCL12-driven ERK1/2 phosphorylation (Figure 5). These results support the involvement of K-Ras in CXCL12-driven ERK phosphorylation in pancreatic cancer cells. Since the reduction of ERK1/2 phosphorylation after KRAS knockdown could be attributed to oncogene addiction due to a dysregulated signaling pathway [29], we tested for this condition by exposing cells to EGF after KRAS knockdown and found that our results are unique to CXCL12 when compared to EGF and suggest the absence of an oncogene addiction phenotype in cells harboring mutant KRAS (Figure 5).

### Signaling through CXCR4 and CXCR7 is β-arrestin-2-dependent

**Figure 6:**
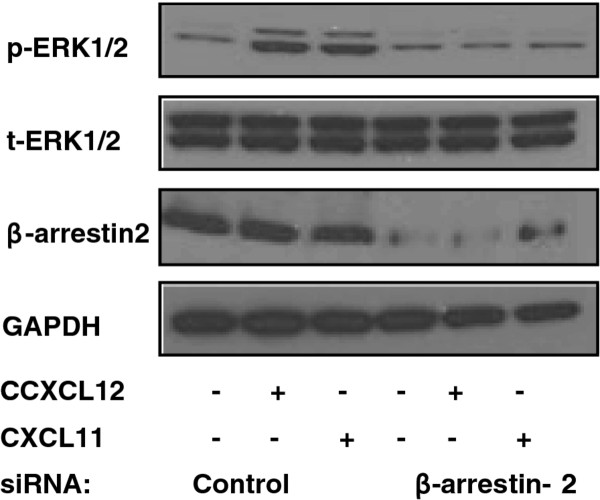
**β-arrestin-2's role in CXCR4 and CXCR7-driven ERK phosphorylation**. PANC-1 cells were transfected with control or β-arrestin-2 siRNA (100 nM) using RNAiMAX (10 μl). Cells were serum-starved and then exposed to CXCL12 (100 ng/ml) or CXCL11 (200 ng/ml) for 15 min. The immunoblot shows β-arrestin-2 and phospho-ERK1/2 expression with total ERK1/2 and GAPDH serving as loading controls.

We sought to determine whether β-arrestin-2 is required for CXCR4 and CXCR7 signaling to the MAPK pathway. We treated cells with both CXCL12 and CXCL11. Down-regulation of β-arrestin-2 expression using siRNA attenuated CXCL12 and CXCL11-driven ERK phosphorylation indicating that β-arrestin-2 mediates CXCR4 and CXCR7 signaling to the MAPK pathway (Figure 6).

### CXCL12 drives an increase in cell proliferation

To determine if CXCL12 exposure results in changes in pancreatic cancer cell proliferation, we exposed cells to CXCL12 and CXCL11 for 72 h. We observed increased cell proliferation. To ascertain which receptor(s) mediated this increase in cell proliferation, we performed CXCR4 and CXCR7 knockdown and once again examined proliferation after 72 h. In the PANC-1 cell line CXCR4 knockdown did not block CXCL12-mediated changes in proliferation, but CXCR7 knockdown did (Figrue 7). Opposite results were obtained for the FG cell line. These results indicate that both CXCR4 and CXCR7 can mediate CXCL12-driven proliferation. The mechanism by which this occurs still needs to be elucidated.

**Figure 7:**
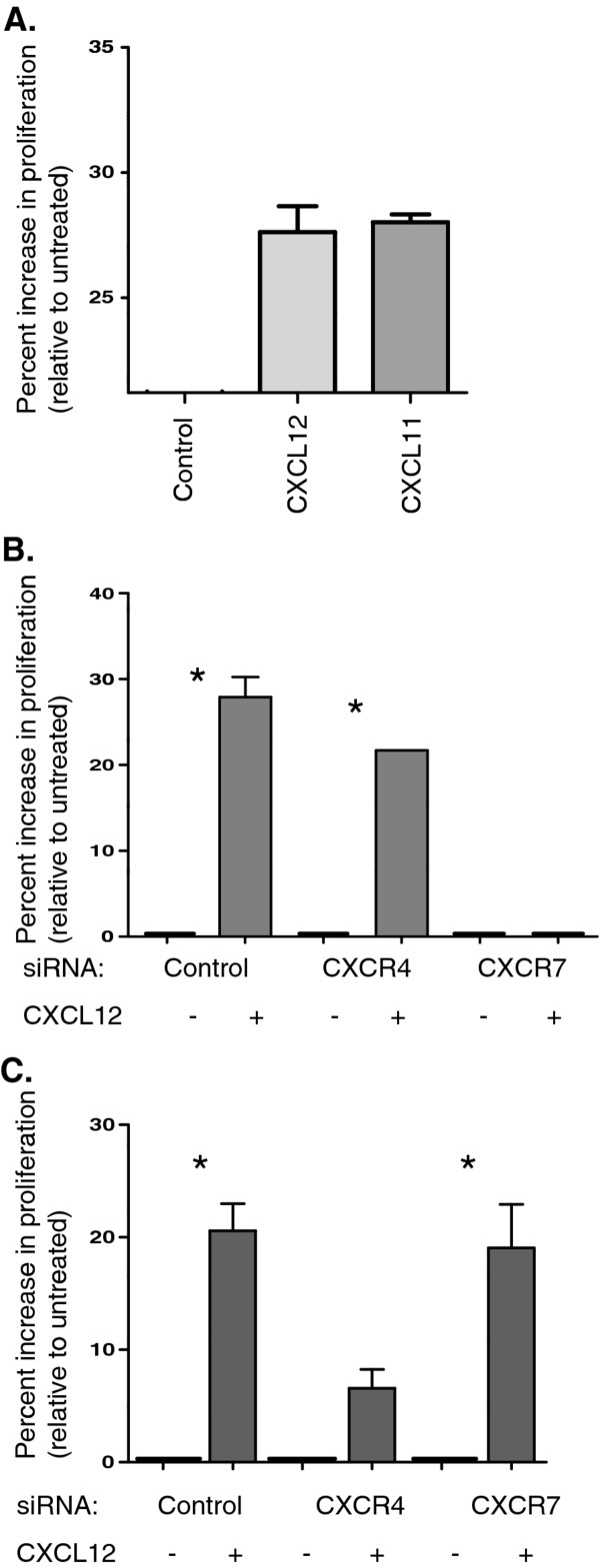
**The effect of CXCL12 on proliferation in pancreatic cancer cells**. A quantitative ATP-based proliferation assay was performed on PANC-1 cells a) treated with CXCL12 and CXCL11 for 72 h.. b) PANC-1 and c) FG cells were transfected with control, CXCR4, or CXCR7 siRNA (100 nM) prior to 72 h of exposure with CXCL12 (200 ng/ml). Proliferation levels presented were normalized to each untreated baseline level, relative increases are depicted. The mean absorbance ± one SD was plotted for each treatment group. * designates *p *< 0.05.

## Discussion

Chemokines regulate the chemotactic responses of cells that are essential for organogenesis and immunity through the orchestration of cell movement from one location to another [30-32]. Cancer cells have misappropriated these regulatory mechanisms to stimulate their own growth, invasion, and metastasis. Numerous studies now implicate chemokines and their corresponding receptors in the invasive phenotype of many cancers [1]. In particular, the CXCL12-CXCR4 axis has been well studied in gastrointestinal malignancies, but recent reports suggest that downstream effects once attributed to CXCR4 may also be secondary to CXCR7, an alternate or second receptor for CXCL12. As a result, we examined CXCL12 activity in pancreatic cancer cell lines. Among several novel discoveries in this investigation, we identified high frequency of CXCR4/CXCR7 co-expression in human pancreatic cancer tissues and cell lines.

Chemokine CXCL12 is characteristically expressed in select tissues,[2] but may also be expressed via an autocrine feedback loop mechanism in pancreatic cancer cells [7]. As such, the resources for CXCL12 to activate CXCR4 or CXCR7-mediated signaling pathways are present. Since our studies target pancreatic cancer, we examined CXCL12 signaling within the framework of K-Ras. The gene encoding KRAS is frequently mutated in patients with pancreatic cancer which results in a gain-of-function that may contribute to the pathogenesis and progression of this cancer [33,34]. Here, we made several novel observations regarding K-Ras. First, we discovered that pancreatic cancer cells co-expressing CXCR4/CXCR7 had increased levels of ERK phosphorylation and K-Ras activity when exposed to CXCL12. These CXCL12-induced increases in K-Ras activity were not observed with other ligands in endometrial and pancreatic cancer cells harboring mutant KRAS [35,36]. Our results, therefore, suggest that CXCL12 may hyperactivate K-Ras activity levels even though there is baseline mutant-derived K-Ras activity. Second, we observed that CXCR4, rather than CXCR7, was the receptor that regulated this response. GPCRs can signal through a canonical or non-canonical pathway, activation of K-Ras may occur via a CXCR4-mediated activation of the canonical GPCR pathway [37-39]. CXCR7 on the other hand signals through the non-canonical pathway [39], which explains why CXCR7 activation leads to ERK1/2 phosphorylation but not an increase in K-Ras activity. Β-arrestin-2 is an important member of either pathway, and we show that its knockdown blocks ERK phosphorylation. Third, we determined that CXCL12-driven increases in cancer cell proliferation can occur through either receptor and hence either signaling pathway.

Specific inhibitors to CXCR4 and CXCR7 are currently unavailable for clinical use. AMD3100 was believed to selectively bind and antagonize CXCR4 activity [40,41]. Derivatives of AMD3100 are also under investigation for their effects on cancer cells [42]. A recent study has demonstrated that AMD3100 specifically binds to and activates CXCR7 [41]. Therefore, in contrast to its antagonism of the CXCL12-CXCR4 interaction, AMD3100 positively modulates CXCL12 effects and binding to CXCR7.

## Conclusion

In summary, we report that CXCR4 and CXCR7 are co-expressed with high frequency in human pancreatic cancer specimens and cell lines. CXCR4 and CXCR7 signaling is β-arrestin-2-dependent and controls CXCL12 signals to the MAPK pathway. CXCL12 activates both canonical and non-canonical GPCR pathways in pancreatic cancer cell lines. This has functional significance in that we show signaling through either pathway leads to an increase in cell proliferation upon exposure to CXCL12. Hence our study suggests that efforts to therapeutically target CXCL12 signaling in pancreatic cancer should be focused at the level of the ligand to account for both CXCR4 and CXCR7 activity.

## Competing interests

The authors declare that they have no competing interests.

## Authors' contributions

JK: made substantial contributions to conception and design of these studies as well as analysis and interpretation of data, drafting the manuscript and revising it critically; WL, JL: acquisition of data and drafting the manuscript; AL: analysis and interpretation of data and drafting the manuscript; ELH: analysis and interpretation of data and revision of the draft. All authors read and approved the final manuscript.
